# Grading amino acid properties increased accuracies of single point mutation on protein stability prediction

**DOI:** 10.1186/1471-2105-13-44

**Published:** 2012-03-22

**Authors:** Jianguo Liu, Xianjiang Kang

**Affiliations:** 1 Life Sciences School, Hebei University, Baoding, Hebei 071002, People's Republic of China

## Abstract

**Background:**

Protein stabilities can be affected sometimes by point mutations introduced to the protein. Current sequence-information-based protein stability prediction encoding schemes of machine learning approaches include sparse encoding and amino acid property encoding. Property encoding schemes employ physical-chemical information of the mutated protein environments, however, they produce complexity in the mean time when many properties joined in the scheme. The complexity introduces noises that affect machine learning algorithm accuracies. In order to overcome the problem we described a new encoding scheme that graded twenty amino acids into groups according to their specific property values.

**Results:**

We employed three predefined values, 0.1, 0.5, and 0.9 to represent 'weak', 'middle', and 'strong' groups for each amino acid property, and introduced two thresholds for each property to split twenty amino acids into one of the three groups according to their property values. Each amino acid can take only one out of three predefined values rather than twenty different values for each property. The complexity and noises in the encoding schemes were reduced in this way. More than 7% average accuracy improvement was found in the graded amino acid property encoding schemes by 20-fold cross validation. The overall accuracy of our method is more than 72% when performed on the independent test sets starting from sequence information with three-state prediction definitions.

**Conclusions:**

Grading numeric values of amino acid property can reduce the noises and complexity of input information. It is in accordance with biochemical concepts for amino acid properties and makes the input data simplified in the mean time. The idea of graded property encoding schemes may be applied to protein related predictions with machine learning approaches.

## Background

Protein thermodynamic stability change upon single point mutations is a crucial problem that affects most protein engineering and molecular biology researches. Significant numbers of different prediction methods have been developed to predict the protein stability free energy change (ΔΔG) in last decades. While energy function-based approaches and statistical analysis were employed to compute the stability free energy change [1-14], machine learning approaches attracted more attention for increasing number of available experimental thermodynamic data in the ProTherm database [15-21]. Given the tertiary structure available, structure information based approaches generally performed better than sequence information based approaches in machine learning approaches [19]. The number of known protein structures, however, is less than one percent (0.45%) of the number of known protein sequences. Current UniProtKB/TrEMBL Release, 2011_08 of 27-Jul-2011, contained 16,504,022 entries of protein sequences while there were only 75,105 structures in PDB till 5 p.m., Tuesday Aug 09, 2011. Most of the available information about proteins is still restricted in their sequence information. Sequence-based protein stability prediction methods attracted more research interests [1-7,15-19].

Sequence-based protein stability prediction methods usually captured the mutation site environments with sliding window strategy with fixed length of the protein sequence that centred on the target residue. The encoding schemes of the sliding window strategy can be grouped into two categories. The first is the sparse encoding schemes that represent each amino acid with twenty distinct input units [16-19]. The second is the amino acid property encoding schemes, which integrate the physical-chemical properties of amino acids into machine learning input information [15]. Rather than representing amino acids with 20 characters, the property encoding schemes employ amino acid physical-chemical properties and usually perform better. There are 20 different numbers that represent each property in the property encoding schemes. If 15 properties were used, there would be 300 different values for each input node. Suppose 31 is the sliding window length, there would be 9,300 possible combinations for each vector. Too much information would be noises to machine learning algorithm.

A possible way to improve the classification task is to try to insert more information in the input code and simultaneously try to refine the quality of the discriminated features. Although each amino acid property can take different numbers, from the physical-chemical point of view, they can be partitioned into three groups: strong, middle or weak group. For example, each amino acid's hydrophobicity can be strong, middle or weak hydrophobicity. If we reduce the number of values for each property to 3, the input information to the algorithm would be much simplified.

Here we developed a property grading method to differentiate the amino acids and reduce the noises of the amino acid properties. We found that the property grading method performed better with the traditional cross-validation test and the current independent test sets.

## Results and discussions

### Three-state prediction definitions

There were 'two-state predictions' and 'three-state predictions' in the protein stability prediction field. In two-state predictions, prediction results were presented as stability "increase" or "decrease"; while in the three-state predictions, the results were presented as stability "increase", "neutral" or "decrease". Although the accuracy scores with two-state predictions usually showed higher, three-state predictions are more reasonable in molecular biology point of view. We adopted Capriotti's 'three-state prediction' definition [19] for all of our experiments.

### Cross validation results with different encoding schemes

Cross validations with one dataset were believed to be the strictest approach to evaluate different encoding schemes. To avoid similarity sequences appearing in both the training and test set at the same time, the sequences were blasted themselves with the dataset sequence database and grouped with their similarities. The sequences with similarity > 25% in blast results were clustered into groups. The groups were randomly selected to a test set. The corresponding training set sequences came from the dataset sequences that were not in the test set. After implementing different encoding schemes and training-test procedures, twenty round cross-validation prediction accuracies were averaged for each encoding scheme.

It is generally held that amino acid physical-chemical property encoding is better than sparse encoding (arbitrary numeric representation of amino acids) because amino acid properties take intrinsic meanings of nature. However, there could be two problems in the property encoding schemes. The first problem could come from the property components to be used. When only one property was adopted, such as hydrophobicity property (K-D in Table 1), the total effects of the prediction could not reach high accuracies. The protein secondary structure propensity factors take information from the protein structure and are expected to be helpful in the input information. However, when they were used alone, we could hardly get good performance either (HEC in Table 1). We used physical-chemical 11-factors encoding which showed almost the same results with the sparse encoding. The sparse encoding scheme (sparse in Table 1) was used as the control in our experiment. When physical-chemical properties and structural propensities combined together, better performance was achieved. AAproperty15 showed a good example of such combinations of the amino acid properties. The overall accuracy (Q_3_) of amino acid property encoding scheme (AAproperty15 in Table 1) was 3% higher than that of sparse encoding schemes. On the other hand, however, it is not true that the more property factors the better. We ever tried as many as 48 factors from aaindex [22] in the encoding scheme and the results showed no improvement to the prediction accuracies (data not shown).

**Table 1:** Cross-validation performance of the sequence-based SVM method of different encoding schemes

Encoding scheme	**Q**_**3**_	MCC	Q(N)	Q(+)	Q(-)	Specificity (N)	Specificity (+)	Specificity (-)	PPV (P (N))	PPV (P (+))	PPV (P (-))	NPV (N)	NPV (+)	NPV (-)	MCC (C(N))	MCC (C(+))	MCC (C(-))
Capriotti^¶^	56.00	0.27	48.00	54.00	54.00	62.00	44.00	44.00	-	-	-	-	-	-	0.17	0.29	0.29
Sparse	56.81	0.28	58.85	54.12	53.51	61.34	79.31	81.10	65.35	65.11	62.31	62.40	75.16	72.37	0.21	0.31	0.33
11-factors	56.92	0.28	59.07	52.32	51.27	63.56	80.77	82.87	68.29	65.46	60.17	63.32	72.32	71.37	0.19	0.32	0.33
HEC	56.91	0.29	58.32	50.56	52.47	66.55	81.39	80.34	65.28	65.43	63.45	65.79	74.56	71.57	0.21	0.32	0.32
K-D	55.98	0.25	57.81	51.64	49.73	63.72	78.29	81.57	63.54	62.14	63.30	66.57	73.22	71.11	0.20	0.34	0.31
AAproperty15	59.57	0.31	61.72	56.13	57.40	60.96	79.57	81.48	68.16	65.89	67.87	65.02	76.83	76.71	0.30	0.35	0.34
AAproperty15Grade	63.63	0.36	64.15	58.23	57.62	61.95	80.35	82.07	69.81	62.52	69.12	67.18	78.31	78.96	0.34	0.39	0.36

All numbers except MCC represent per cent values. +, - and N: the indexes are evaluated for increasing, decreasing or neutral protein free energy stability change, respectively according to the classification described in section 2 of Results and Discussions; for the definition of the different indexes see the Scoring the performance in Methods. ^¶ ^data from Capriotti [19]

The second problem that embarrassed the property encodings could come from the noises and the data complexities from the input factors. Grading the property numeric values can reduce the noises from the input factors and achieve better performances. When the properties were graded into three classes and represented by three distinct numbers (AAproperty15Grade in Table 1), the predictions presented better results. Q_3 _of AAproperty15Grade was 4% higher than that of non-graded schemes (AAproperty15 in Table 1). In general, the graded property encoding scheme achieved 7% better than sparse encoding scheme in prediction accuracies. Matthew's correlation coefficient (MCC) showed improvements also. With the graded property encoding scheme, the sequence based method can even be competitive with the structure based approach (Q_3 _61% and Mcc 0.35 [19]) in the three-state mutation stability predictions.

### Test results on independent test datasets

Dataset DBSEQ_Sep05 was used to make our prediction model. When evaluating its performance, the chosen independent test sets were blasted against the DBSEQ_Sep05 sequence database. Mutation samples were deleted from the chosen independent test set that the sequences share similarities bigger than 25% with the ones in the Additional file 1: DBSEQ_Sep05 dataset. 1132 sequence similarity mutations, for example, were deleted from the Potapov data set (2153 mutation samples in 79 proteins), and the resulted independent test set Additional file 2: *clean.Potapov *retained only 1021 mutations in 50 protein chains. The statistics and explanation of the clean independent test sets were shown in Additional file 3: Table S1 and S2.

Table 2 showed the prediction accuracies when predicted the clean independent test sets with the graded property encoding DBSEQ_Sep05 model. Average accuracy of Q_3 _72.55% explained the advantage of graded-property encoding scheme, which is highest accuracy that can be found in the literature with three-state predictions.

**Table 2:** Performance on independent datasets

Test set	**Q**_**3**_	MCC	Q(N)	Q(+)	Q(-)	Specificity (N)	Specificity (+)	Specificity (-)	PPV (P (N))	PPV(P (+))	PPV (P (-))	NPV (N)	NPV (+)	NPV (-)	MCC (C(N))	MCC (C(+))	MCC (C(-))
clean.TEST_ May11	71.82	0.51	80.65	63.21	62.35	66.90	92.10	92.17	71.19	72.75	72.72	77.13	88.34	88.09	0.48	0.58	0.51
clean.S1615	72.71	0.54	79.68	66.79	65.62	70.26	91.45	92.14	72.73	71.95	73.15	76.98	89.21	88.94	0.50	0.60	0.55
clean.S388	74.49	0.56	81.84	67.70	66.59	70.29	92.67	93.19	73.16	75.37	76.28	79.31	89.57	89.29	0.52	0.63	0.57
clean.PoPMuSiC	72.17	0.53	76.57	68.40	66.94	72.04	90.48	91.18	72.94	70.57	71.34	75.39	89.60	89.22	0.48	0.59	0.56
clean.Potapov	71.58	0.52	79.30	64.47	63.80	68.34	91.77	91.77	71.08	72.41	72.11	76.97	88.42	88.21	0.48	0.58	0.52
Average	72.55	0.53	79.61	66.11	65.06	69.57	91.69	92.09	72.22	72.61	73.12	77.16	89.03	88.75	0.49	0.60	0.54

For notation see Table 1. Independent test set details and statistics see Table S1 and S2

### ROC comparisons

**Figure 1:**
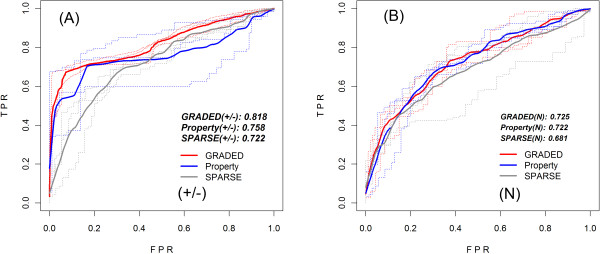
**ROC curves for different encoding schemes of the sequence-based predictor**.

When the sparse encoding and amino acid property encoding schemes are considered, a slight improvement of amino acid property encoding scheme is detected. This can be seen from both the stabilizing/destabilizing and neutral mutation ROC curves (Figure 1). In the case of comparing graded amino acid property encoding vs. amino acid property encoding, the AUC of graded amino acid property is evident bigger than that of amino acid property encoding scheme in the stabilizing/destabilizing mutations (Figure 1A).

ROC curves for the three encoding schemes. The cross-validation True Positive Rate (TPR) versus the False Positive Rate (FPR) are plotted for the sparse, the property and the graded property encoding schemes. In part (A), the ROC curves of the three encoding schemes are relative to the stabilizing and destabilizing mutations, while in part (B), the curves represent neutral mutations. The solid lines are the average values for independent tests of the scheme; and the dashed lines are the test instances to show the distributions of the test values. The vertical bars represent standard errors

## Conclusions

Physical-chemical properties of amino acids take intrinsic meanings of nature, which make proteins present common characteristics of life. Numerical representations of the properties come from the real world experiments and are the results of balanced multiple physical forces. The amino acid physical-chemical property encodings, if well used in protein related predictions, should be better approaches than factitious encodings like sparse encoding, arbitrary numeric representations of amino acids.

The graded physical-chemical property approach discriminates amino acids into strong, middle, or weak groups according to their specific property values. It is in accordance with biochemical concepts for amino acid properties, and makes data simplified in the mean time. The idea of grading properties may be applied to protein related predictions with machine learning approaches.

## Methods

### Data descriptions

Experimental data in the ProTherm database [21] are affected by errors. When the value of the free energy change is close to 0 and the associated error is considered, for one single measure the sign of ΔΔG can change from decreasing to increasing and vice versa. The ΔΔG threshold value for mutation classification is limited to the value of standard errors reported in experimental works. In accordance with Capriotti's criteria [18], we used |1.0| kcal/mole as the threshold for classifications. According to its experimental ΔΔG value each mutation sequence is grouped into one of the following three classes:

i) destabilizing mutation, when ΔΔG < -1.0 kcal/mole;

ii) stabilizing mutation when ΔΔG > 1.0 kcal/mole;

iii) neutral mutations when -1.0 ≤ ΔΔG ≤ 1.0 kcal/mole.

The data set compiled by Capriotti [19], named DBSEQ_Sep05 data set, was used to develop our models. S1615, S388 data sets [16], PoPMuSiC [9], Potapov-DB dataset [8] and TEST_May09 were chosen for independent performance comparisons.

DBSEQ_Sep05 data set contained 1623 different single point mutations and related experimental data for 58 different proteins. Among these mutations, there were 138 stabilizing mutations, 663 destabilizing mutations, and 822 neutral mutations. The samples for three classes were quite unbalanced and they would lead bias in the model training in machine learning procedures. From the point of view of basic thermodynamics, a protein and its mutated form should be endowed with the same free energy change, irrespectively of the reference protein (native or mutated). Hence, we can assume that the module of free energy change is the same in going from one molecule to the other and that what changes is only the ΔΔG sign. The problem of the asymmetric abundance of the three classes was overcome by reversing the mutation ΔΔG sign, we doubled the stabilizing/destabilizing samples and got 801 stabilizing, 801 destabilizing, 822 neutral mutation samples.

S1615 data set was compiled from an earlier version of the ProTherm release and thus included less data when compared with data set DBSEQ_Sep05. The S388 data set is a subset of S1615, containing only physiological condition data derived under temperatures from 20.8°C to 40.8°C and pH values from 6 to 8.

PoPMuSiC dataset was compiled by PoPMuSiC-2.0 [9] with 2648 different point mutations in 131 proteins. Only mutations in globular proteins were considered, PoPMuSiC dataset was believed to be non-redundant data set itself for defining as a weighted average of all available ΔΔG values in favor of normal experiment conditions including temperature and pH when mutants taking variant ΔΔG values.

Potapov-DB dataset [8] contained 2155 mutations in 79 proteins. Single- and multi-site mutations were considered. Potapov-DB removed redundant data by averaging free energy change (ΔΔG) of the mutants when multiple data available.

All the above datasets were compiled with different constraints and conditions by different people. The datasets could be non-redundant themselves; however, they were searched from the same ProTherm database and could share some homologues to each other. In order to give a fair and controllable independent assessment of our model, we built a new dataset TEST_May11 from the updated ProTherm database (from September 2005 to May 2011) with Capriotti's[19] searching constraints: only single point mutations; reversible experiments; and the ΔΔG value with known experimental conditions (temperature and pH). The training set DBSEQ_Sep05 was built early by Capriotti in September 2005. TEST_May11 contains 1004 mutations in 51 proteins with 375 destabilizing mutations (ΔΔG < -1.00 Kcal/mol), 61 stabilizing mutations (ΔΔG > +1.00 Kcal/mol) and 568 neural mutations (-1.00 < = ΔΔG < = +1.00 Kcal/mol).

### Data sets clean up

To avoid the introduction of mutations that share similarity with those of the DBSEQ_Sep05 training set, the independent data sets *TEST_May11, S388, Potapov, PoPMuSic*, and *S1615 *were blasted against the DBSEQ_Sep05 seq58-protein database. Mutation samples were deleted from the test set that share sequence similarities (identity > 25%) with mutation site in

*q.start ~ q.end *sequence region in the blast results (Additional file 4: *blast.independent175.against.seq58*). For example, 934 redundant mutations were deleted from PoPMuSic data set (2648 mutation samples in 134 proteins), and the resulted data set *clean.PoPMuSic *retained only 1712 mutations in 109 protein chains. After removing all these sequence similarity mutation samples, we got the "clean" test sets: Additional file 2*: clean.TEST_May11, clean.S388, clean.Potapov, clean.PoPMuSic*, and *clean.S1615*. The test files can be found in the supplementary materials of the paper. The statistics and explanation of the clean test sets were shown in Additional file 3: Table S1 and S2. The clean datasets were used to evaluate our prediction model.

### Balancing mutation samples

Experimental data in the ProTherm database are intrinsically non symmetric and unbalanced, with destabilizing mutations outnumbering stabilizing ones. Unbalanced training samples would result in poor accuracy on the minority/positive samples in machine learning such as SVM. This is because the class-boundary learned by the SVM is skewed towards the majority/negative class, which may lead to many positive examples being classified as negative (false negatives). From the point of view of basic thermodynamics, a protein and its mutated form should be endowed with the same free energy change. The problem of the asymmetric abundance of the three classes can be solved by reversing mutation (namely the mutation that transforms back the mutant into the original protein) by considering the value of the experimental measure with the opposite sign (-ΔΔG).

### 20-fold Cross validation test

The data set DBSEQ_Sep05 was adopted in our experiments to make cross validation tests for different encoding schemes. In order to make similarity sequences in the same partition, DBSEQ_Sep05 sequences were blasted themselves with the DBSEQ_Sep05 sequence database. The results were shown in Additional file 5: *blast.DBSEQ_Sep05*. With similarity > 25%, the mutation samples were clustered into 58 groups. The groups were random selected and joined to make a test set. The corresponding training set to the test set was produced from the data set DBSEQ_Sep05 by finding entries that were not in the test set. The groups, test sets and complementary training sets were explained in the Additional file 6: *blast.group.DBSEQ_Sep05 *and Additional file 7: *TrainTestSet.description*. The "serials" in the explanations corresponded to the sample entries in the Additional file 1: *DBSEQ_Sep05.txt *dataset. The "group" was the GROUP number defined in Additional file 6: *blast.group.DBSEQ_Sep05*. The test/training sets were then balanced with reversing the ΔΔG sign with the criteria of ΔΔG < -1.0 or ΔΔG > 1.0. The encoding schemes applied to each test/training set afterwards. Each round of the cross validation test consisted of twenty iterations of the training/test procedure. Twenty round cross validations were accomplished for each encoding scheme, and the test accuracies were averaged for the scheme.

### The predictors

The LibSVM package 2.82 [23] was used for SVM training and prediction. The radial basis function (RBF kernel = exp[-G || x*_i _*- x*_j _*||^2^]) was used as kernel function in the experiment. The cost parameter *C *and kernel parameter *g *were optimized with the package built-in tool *grid*, which would take several hours for each training subset. The optimized *C *and *g *values were determined by *grid *results and were different from subset to subset depending on the data distributions of the specific random partitions. *C *values varied from 2 to 32768 and *g *values from 0.0078125 to 2.0 from our lab record and theoretically they could go even farther. The optimized *C *and *g *were used to train LibSVM with the training subset and a model resulted. The model was used to predict protein stabilities with the corresponding test subset. A given single point protein mutation was classified in one of the three classes: stabilizing, destabilizing and neutral. The classes were represented by three labels: "0" for stabilizing mutations (ΔΔG > 1.0 kcal/mole), "1" for destabilizing mutations (ΔΔG < -1.0 kcal/mole) and "2" for neutral mutations (-1.0 ≤ ΔΔG ≤ 1.0 kcal/mole).

### Input vectors and encoding schemes

One of important steps in machine learning approaches is to encode the raw materials data into format data that can be recognized by machines. To encode the mutated position and the surrounding environments of the position into vectors, we employed the deleted residue, the introduced residue, the environment window amino acids around the mutated position, experimental pH and temperature, *etc*.

#### Sparse encoding scheme

The most widely-used representation of an amino acid sequence in bioinformatics modelling is the "sparse encoding" scheme [19]. The input vector consists of 42 values. The first 2 input values account respectively for the temperature and the pH at which the stability of the mutated protein was experimentally determined. The next 20 values (for 20 residue types) explicitly define the mutation, setting to -1 the element corresponding to the deleted residue and to 1 the new residue (all the remaining elements are kept equal to 0). The last 20 input values encode the residue environment: each of the 20 input values is the number of the encoded residue type found inside a symmetrical window centred at the mutated residue, spanning the sequence towards the left (N-terminus) and the right (C-terminus), for a total length of 31 residues [19].

#### 11-factor encoding scheme

Sparse encoding scheme represents amino acids with different numbers and the numbers themselves having no relation with the physicochemical properties of the amino acids. Leucine, for instance, have similar polarity with isoleucine but quite different from glutamic acid. However, Leu, Ile and Glu have same status in sparse encoding scheme by taking different numbers. Sparse encoding scheme does not account for any similarity in physicochemical properties between amino acids. Liu W. *et al. *successfully used amino acid property encoding schemes with support vector machines [24]. They extracted 17 amino acid physicochemical parameters from AAindex, after eliminating related properties with correlation coefficient factor (*r*^2 ^> 0.8), and got a good performance with 11 factors, which were linearly scaled to the range of [0,1] from the raw data. We used their 11-factor encoding scheme in our experiment.

#### HEC encoding scheme

Chou-Fasman's amino acid propensity parameters to protein secondary structure conformation, namely helix propensity (He), sheet propensity (Ee), and coil propensity (Ce) [25], were recalculated with modern non-redundant protein secondary structure dataset

CB513 [26] and RS126 [27]. To test the amino acid conformation propensity properties in our experiment, the propensity parameters were transformed into the range [0,1] with 1/(1 + *e*^-x^) formula.

#### K-D encoding scheme

Amino acid hydrophobicity was believed to be one of the most important properties to maintain the protein tertiary structure. Kyte and Doolittle's hydrophobicity scale [28] was used to test a single amino acid property effect in prediction. We transformed the Kyte and Doolittle's data into the range [0,1] with 1/(1 + *e*^-x^) formula, and named as K-D encoding scheme.

#### Property encoding scheme (AAproperty15)

The 11-factor amino acid properties were combined with the amino acid secondary structure conformation propensity parameters He, Ee, and Ce. To emphasize hydrophobicity's proportion in its influence in protein structure, Kyte-Doolittle hydrophobicity scale was added to the encoding scheme also. A list of 15 factors was obtained (Table 3). We named the encoding scheme as "AAproperty15".

**Table 3:** The amino acid property scores used in the AAproperty15 encoding scheme

AA	Steric parameter	Hydrogen Bond Donors	Hydrophobicity scale	Hydrophilicity scale	Average Accessible surface area	van der Waals Parameter R0	van der Waals Parameter Epsilon	Free Energy of solution in water	Average side chain orientation Angle	Polarity	Isoelectric point	He	Ee	Ce	KDe
A	0.510	0.169	0.471	0.279	0.141	0.294	0.000	0.262	0.512	0.000	0.404	0.811	0.667	0.700	0.858
R	0.667	0.726	0.321	1.000	0.905	0.529	0.327	0.169	0.372	1.000	1.000	0.777	0.691	0.719	0.011
N	0.745	0.390	0.164	0.658	0.510	0.235	0.140	0.313	0.116	0.065	0.330	0.691	0.655	0.790	0.029
D	0.745	0.304	0.021	0.793	0.515	0.235	0.140	0.601	0.140	0.956	0.000	0.725	0.624	0.783	0.029
C	0.608	0.314	0.760	0.072	0.000	0.559	0.140	0.947	0.907	0.028	0.285	0.661	0.804	0.737	0.924
Q	0.667	0.531	0.178	0.649	0.608	0.529	0.140	0.416	0.023	0.068	0.360	0.778	0.683	0.722	0.029
E	0.667	0.482	0.092	0.883	0.602	0.529	0.140	0.561	0.163	0.960	0.056	0.812	0.652	0.707	0.029
G	0.000	0.000	0.275	0.189	0.103	0.000	0.000	0.240	0.581	0.000	0.401	0.619	0.665	0.821	0.401
H	0.686	0.554	0.326	0.468	0.402	0.529	0.140	0.313	0.581	0.992	0.603	0.715	0.754	0.732	0.039
I	1.000	0.650	1.000	0.000	0.083	0.824	0.308	0.424	0.930	0.003	0.407	0.734	0.844	0.658	0.989
L	0.961	0.650	0.734	0.081	0.138	0.824	0.308	0.463	0.907	0.003	0.402	0.792	0.768	0.664	0.978
K	0.667	0.692	0.000	0.568	1.000	0.529	0.327	0.313	0.000	0.952	0.872	0.755	0.701	0.731	0.020
M	0.765	0.612	0.603	0.171	0.206	0.765	0.308	0.405	0.814	0.028	0.372	0.794	0.763	0.665	0.870
F	0.686	0.772	0.665	0.000	0.114	0.853	0.682	0.462	1.000	0.007	0.339	0.747	0.807	0.676	0.943
P	0.353	0.372	0.012	0.198	0.411	0.588	0.271	0.000	0.302	0.030	0.442	0.629	0.608	0.835	0.168
S	0.520	0.172	0.155	0.477	0.303	0.206	0.000	0.240	0.419	0.032	0.364	0.681	0.711	0.773	0.310
T	0.490	0.349	0.256	0.523	0.337	0.235	0.140	0.313	0.419	0.032	0.362	0.667	0.780	0.748	0.332
W	0.686	1.000	0.681	0.207	0.219	1.000	1.000	0.537	0.674	0.040	0.390	0.759	0.815	0.661	0.289
Y	0.686	0.796	0.591	0.477	0.454	0.853	0.682	1.000	0.419	0.031	0.362	0.721	0.813	0.692	0.214
V	0.745	0.487	0.859	0.036	0.094	0.647	0.234	0.369	0.674	0.003	0.399	0.714	0.864	0.655	0.985

#### Graded property encoding scheme (AAproperty15Grade)

Comparing with sparse encoding, complexity may be the problem introduced by property encoding scheme. For each property, amino acids take 20 different values. 15 properties and window length of 31 can make 9300 values. In addition to encoding the deleted residue, the new residue, temperature and pH, property encoding scheme introduced complexity while there are numerous benefits and advantages associated with the scheme.

According to a specific physicochemical property, all amino acids can usually be grouped into strong, middle, or weak classes. For hydrophobicity, we can have strong hydrophobic, middle hydrophobic, and weak hydrophobic amino acids. The amino acid numeric representations of each property can be partitioned into three groups if we define two numeric thresholds.

Rather than direct using the amino acid property numeric values in the encoding scheme, we define three distinct numbers to represent the strong, middle, or weak classes. When the numeric representation is less than the lower limit, we represent the amino acid as 0.1; when greater than the upper limit, we represent the amino acid as 0.9; when the numeric is equal or greater than lower limit but equal or less than upper limit, the amino acid is represented as 0.5, as shown in Equation 1. The lower limit and the upper limit are arbitrary numbers that can partition 20 amino acids evenly into three groups according to the distribution of the property numeric values.

(1) $$S_{i}^{a}=\left\{\begin{array}{c} \text{ 0}\text{.1}\;\;if\;\;P_{i}^{a}\;<\;\;L_{i}\\ \text{ 0}\text{.5}\;\;if\;Li\leq P_{i}^{a}\leq U_{i}\\ \text{ 0}\text{.9}\;\;if\;P_{i}^{a}\;>U_{i} \end{array}\right)$$

Where $S_{i}^{a}$ is the score used in the coding scheme, $P_{i}^{a}$ is the numeric value of property *i *of amino acid *a*, *L_i _*is the lower limit of property *i*, and *U_i _*is the upper limit of property *i*.

For each property, two thresh-holds partition twenty amino acids into three classes: weak, middle, or strong class. Each amino acid took one out of three rather than one out of twenty different numbers for each property. The complexity and noises can be much reduced in this way. Table 3 showed fifteen amino acid property encoding values and Table 4 showed scores used in the graded encoding schemes, which were derived from Table 3 with two thresholds. The thresholds used were arbitrary and the intention was to get as equal number of the amino acids in each group as possible. The thresholds (lower limit/upper limit) are: steric parameter 0.65/0.7; hydrogen bond donors 0.35/0.66; hydrophobicity scale 0.25/0.65; hydrophilicity scale 0.18/0.55; average accessible surface area 0.2/0.5; van der Waals parameter R_0 _0.3/0.7; van der Waals parameter Epsilon 0.1/0.5; free energy of solution in water 0.4/0.55; average side chain orientation angle 0.3/0.7; polarity 0.02/0.5; isoelectric point 0.3/0.401; He 0.72/0.76; Ee 0.67/0.8; Ce 0.7/0.75; and KDe 0.1/0.8.

**Table 4:** The graded amino acid property encoding scheme AAproperty15Grade

AA	Steric	Donors	Hydrophobicity	Hydrophilicity	Accessible	R0	Epsilon	FreeEnergy	Angle	Polarity	Isoelectric	He	Ee	Ce	KDe
A	0.1	0.1	0.5	0.5	0.1	0.1	0.1	0.1	0.5	0.1	0.9	0.9	0.1	0.5	0.9
R	0.5	0.9	0.5	0.9	0.9	0.5	0.5	0.1	0.5	0.9	0.9	0.9	0.5	0.5	0.1
N	0.9	0.5	0.1	0.9	0.9	0.1	0.5	0.1	0.1	0.5	0.5	0.1	0.1	0.9	0.1
D	0.9	0.1	0.1	0.9	0.9	0.1	0.5	0.9	0.1	0.9	0.1	0.5	0.1	0.9	0.1
C	0.1	0.1	0.9	0.1	0.1	0.5	0.5	0.9	0.9	0.5	0.1	0.1	0.9	0.5	0.9
Q	0.5	0.5	0.1	0.9	0.9	0.5	0.5	0.5	0.1	0.5	0.5	0.9	0.5	0.5	0.1
E	0.5	0.5	0.1	0.9	0.9	0.5	0.5	0.9	0.1	0.9	0.1	0.9	0.1	0.5	0.1
G	0.1	0.1	0.5	0.5	0.1	0.1	0.1	0.1	0.5	0.1	0.5	0.1	0.1	0.9	0.5
H	0.5	0.5	0.5	0.5	0.5	0.5	0.5	0.1	0.5	0.9	0.9	0.1	0.5	0.5	0.1
I	0.9	0.5	0.9	0.1	0.1	0.9	0.5	0.5	0.9	0.1	0.9	0.5	0.9	0.1	0.9
L	0.9	0.5	0.9	0.1	0.1	0.9	0.5	0.5	0.9	0.1	0.9	0.9	0.5	0.1	0.9
K	0.5	0.9	0.1	0.9	0.9	0.5	0.5	0.1	0.1	0.9	0.9	0.5	0.5	0.5	0.1
M	0.9	0.5	0.5	0.1	0.5	0.9	0.5	0.5	0.9	0.5	0.5	0.9	0.5	0.1	0.9
F	0.5	0.9	0.9	0.1	0.1	0.9	0.9	0.5	0.9	0.1	0.5	0.5	0.9	0.1	0.9
P	0.1	0.5	0.1	0.5	0.5	0.5	0.5	0.1	0.5	0.5	0.9	0.1	0.1	0.9	0.5
S	0.1	0.1	0.1	0.5	0.5	0.1	0.1	0.1	0.5	0.5	0.5	0.1	0.5	0.5	0.5
T	0.1	0.1	0.5	0.5	0.5	0.1	0.5	0.1	0.5	0.5	0.5	0.1	0.5	0.5	0.5
W	0.5	0.9	0.9	0.5	0.5	0.9	0.9	0.5	0.5	0.5	0.5	0.5	0.9	0.1	0.5
Y	0.5	0.9	0.5	0.5	0.5	0.9	0.9	0.9	0.5	0.5	0.5	0.5	0.9	0.1	0.5
V	0.9	0.5	0.9	0.1	0.1	0.5	0.5	0.5	0.5	0.1	0.5	0.1	0.9	0.1	0.9

### Scoring the performance

Seven indices, total accuracy(sensitivity) (Q3) (Equation 2) and total Matthew's correlation coefficient (MCC) (Equation 3) [29], the accuracy(sensitivity) (Q) (Equation 4), specificity(Equation 5), positive predictive value (PPV) (Equation 6), negative predictive value(NPV) (Equation 7), MCC (Equation 8), were calculated for the assessment of the prediction system.

(2) $$Q_{total}=\frac{\overset{k}{\underset{i=1}{\sum }}p\left(i\right)}{N}$$

(3) $$MCC_{total}=\frac{\overset{k}{\underset{i=1}{\sum }}\left(p\left(i\right)+u\left(i\right)\right)MCC\left(i\right)}{N}$$

(4) $$Q\left(i\right)=\frac{p\left(i\right)}{p\left(i\right)+u\left(i\right)}$$

(5) $$specificity\left(i\right)=\frac{n\left(i\right)}{n\left(i\right)+o\left(i\right)}$$

(6) $$PPV\left(i\right)=\frac{p\left(i\right)}{p\left(i\right)+o\left(i\right)}$$

(7) $$NPV\left(i\right)=\frac{n\left(i\right)}{n\left(i\right)+u\left(i\right)}$$

(8) $$MCC\left(i\right)=\frac{p\left(i\right)n\left(i\right)-u\left(i\right)o\left(i\right)}{\sqrt{\left[p\left(i\right)+u\left(i\right)\right]\left[p\left(i\right)+o\left(i\right)\right]\left[n\left(i\right)+u\left(i\right)\right]\left[n\left(i\right)+o\left(i\right)\right]}}$$

Here, *i *is the any subfamily, *N *is the total number of sequences, *k *is the subfamily number, *p*(*i*) is the number of correctly predicted sequences of subfamily *i*, *n*(*i*) is the number of correctly predicted sequences not of subfamily *i*, *u*(*i*) is the number of under-predicted sequences, and *o*(*i*) is the number of over-predicted sequences, in other words, *p(i) *= TP, *n(i) *= TN, *u(i) *= FN, *o(i) *= FP.

### Multi-class ROCR

Currently, ROCR supports only binary classification [30,31], if there are more than two distinct label symbols, execution stops with an error message. To overcome the binary classification limit of ROCR package, we defined functions *split.class *and *split.probabilities *to split classes and probabilities independently and make the data become *one-against-rest*. We collected the three class probabilities with ROCR built-in function *predict *(probability = TRUE). With *list *function, we then joined the independent data of three classes together and plot ROC curve. The ROC curve can then represent the three class classification. User defined functions can be found in the Additional file 8: *multi.class.functions.rocr*.

## Competing interests

The authors declare that they have no competing interests.

## Authors' contributions

JL contributes extracting data from ProTherm, implementing the predictors and writing the paper. XK contributes in the discussion of the encoding schemes, in the review of the results and also in writing the paper. Both authors read and approved the final manuscript.

## Supplementary Material

Additional file 1 **DBSEQ_Sep05**. The file containing the data for cross-validation tests is available as supplementary material as ASCII files. Click here for file

Additional file 2 **Clean.independent.zip**. The file containing the independent test files with no sequence similarity > 25% to DBSEQ_Sep05 sequences is compressed in zip file and available as supplementary material as ASCII files. The file contains *clean.PoPMuSic, clean.Potapov*, *clean.S388, clean.S1615 *and *clean.TEST_May11 *files, which are described in Table S1 and S2. Click here for file

Additional file 3 **Table S1: independent test set statistics**. Table S2: data descriptions of the independent test set. Click here for file

Additional file 4 **Blast.independent175.against.seq58**. The file containing the blast results of independent test set sequences to DBSEQ_Sep05 sequence database is available as supplementary material as ASCII files. Click here for file

Additional file 5 **Blast.DBSEQ_Sep05**. The file containing the blast results of DBSEQ_Sep05 sequences to DBSEQ_Sep05 sequence database is available as supplementary material as ASCII files. Click here for file

Additional file 6 **Blast.group.DBSEQ_Sep05**. The file containing the cluster results with group similarity > 25% sequences in blast results is available as supplementary material as ASCII files. Click here for file

Additional file 7 **TrainTestSet.description**. The file containing the random group selections and test-training set descriptions used in the cross-validation tests is compressed in zip file TrainTestSet.zip and available as supplementary material as ASCII files. Click here for file

Additional file 8 **Multi.class.rocr.functions**. The file containing the user defined functions used to multi-class ROCR is available as supplementary material as ASCII files. Click here for file
