# Supplementary material for: Grading amino acid properties increased accuracies of single point mutation on protein stability prediction
Source: BMC Bioinformatics. 2012 Mar 22;13:44. doi: 10.1186/1471-2105-13-44 (PMC3820156; doi:10.1186/1471-2105-13-44)
Supplement: Additional file 3 — Table S1: independent test set statistics. Table S2: data descriptions of the independent test set. [file 1471-2105-13-44-S3.doc]

## Table S1: independent test set statistics

| Independent Test Set | | PoPMuSiC | Potapov | S1615 | S388 | TEST_May11 |
| --- | --- | --- | --- | --- | --- | --- |
| Original | Samples | 2648 | 2153 | 1615 | 388 | 1004 |
|  | Proteins^§^ | 134 | 79 | 41 | 17 | 51 |
| Clean up^¶^ | Samples | 1712 | 1021 | 87 | 15 | 366 |
|  | Proteins^§^ | 109 | 50 | 12 | 5 | 18 |

^¶^ deleted mutation samples from original dataset that share sequence similarities (identity > 25%) to DBSEQ_Sep05 dataset with q. start ~ q. end sequence region in the blast results. ^§^ total protein sequence numbers in the test data set. Details can be referenced in “additional files” in the supplementary materials.

## Table S2: data descriptions of the independent test set

| Test set name | Descriptions of the data |
| --- | --- |
| clean.TEST_May11 | 366 mutation samples in 18 protein chains  Complement Data to DBSEQ_Sep05 from TEST_May11 data set  Deleted 638 redundant from TEST_May11 (1004 samples in 51 proteins) |
| clean.S1615 | 87 mutation samples in 12 protein chains  Complement Data to DBSEQ_Sep05 from S1615 data set  Deleted 1528 redundant from S1615 (1615 samples in 41 proteins) |
| clean.S388 | 15 mutation samples in 5 protein chains  Complement Data to DBSEQ_Sep05 from S388 data set  Deleted 373 redundants from S388 (388 samples in 17 proteins) |
| clean.Potapov | 1021 mutation samples in 50 protein chains  Complement Data to DBSEQ_Sep05 from Potapov data set  Deleted 1132 redundants from Potapov (2153 samples in 79 proteins) |
| clean.PoPMusic | 1712 mutation samples in 109 protein chains  Complement Data to DBSEQ_Sep05 from PoPMusic data set  Deleted 934 redundants from PoPMusic (2648 samples in 134 proteins) |
